# In Vivo and In Vitro Study of Immunostimulation by *Leuconostoc lactis*-Produced Gluco-Oligosaccharides

**DOI:** 10.3390/molecules24213994

**Published:** 2019-11-05

**Authors:** Sulhee Lee, In Ho Song, Young-Seo Park

**Affiliations:** 1 Department of Food Science and Biotechnology, Gachon University, Seongnam 13120, Korea; sulhee2340@gmail.com; 2 Research Group of Healthcare, Korea Food Research Institute, Wanju 55365, Korea; 3 Department of Nuclear Medicine, Seoul National University Bundang Hospital, Seoul National University College of Medicine, Seongnam 13620, Korea; 99269@snubh.org

**Keywords:** oligosaccharides, immunostimulation, MAPK signaling pathway, phagocytosis, cyclophosphamide

## Abstract

Glycosyltransferase-producing *Leuconostoc lactis* CCK940 produces CCK- oligosaccharides, gluco-oligosaccharide molecules, using sucrose and maltose as donor and acceptor molecules, respectively. In this study, the immunostimulatory activities of CCK-oligosaccharides on RAW264.7 macrophages and BALB/c mice were evaluated. CCK-oligosaccharides induced the expression of phosphorylated-p38, extracellular-signal-regulated kinase (ERK), and c-Jun N-terminal kinase (JNK) and upregulation of phagocytic activity in RAW264.7 macrophages, suggesting their involvement in mitogen-activated protein kinase (MAPK) signaling pathway and phagocytosis. When CCK-oligosaccharides were administered to mice intraperitoneally injected with cyclophosphamide (CY), spleen indices and expressions of interleukin (IL)-6, IL–10, and tumor necrosis factor-α increased, compared with those in only CY-treated group. These findings suggest that CCK-oligosaccharides can be used as an effective immunostimulating agent.

## 1. Introduction

Some lactic acid bacteria produce oligosaccharides by the enzyme reaction of glucansucrase (EC 2.4.1.5), alternansucrase (EC 2.4.1.140), mutansucrase (EC 2.4.1.5), or levensucrase (EC 2.4.1.10) [1,2,3,4]. In a previous study, *Leuconostoc lactis* CCK940, isolated from Chinese cabbage kimchi, was found to have a high glycosyltransferase activity [5]. It produces gluco-oligosaccharides, namely CCK-oligosaccharides, in large quantities and the production of oligosaccharides using this strain was optimized using response surface methodology. CCK-oligosaccharides have been found to increase the mRNA expression levels of tumor necrosis factor (TNF)-α, interleukin (IL)-1β, IL-6, and inducible nitric oxide synthase in RAW264.7 macrophages [6].

The mammalian immune system is composed of acquired and innate immune systems [7]. The innate immune system is mediated by phagocytes, including macrophages and neutrophils, and is a crucial component of host defense against invading microbial pathogens [8,9]. The activation of macrophages causes the production of inflammatory cytokines and mediators [10,11]. Various polysaccharides and some oligosaccharides, such as chitosan oligosaccharides, galacto-oligosaccharides, xylo-oligosaccharides, and alginate oligosaccharides, have been found to exert immunostimulating effects both in vivo and in vitro [12,13,14,15,16].

Cyclophosphamide (CY) is a well-known alkylating agent [17], and is an important chemotherapeutic drug used in tumor treatment [18]. It is also used in organ transplantation, and to treat autoimmune diseases and cancer patients. CY causes an immunosuppressive effect by damaging DNA of normal cells [19] and is one of the major immunosuppressive medicines used in clinical trials [20]. Therefore, it is unfavorable for the immune system of organisms because immunosuppression can be fatal [18].

In the present study, the mechanism of immnunostimulatory effects of CCK-oligosaccharides was determined by Western blot and real-time polymerase chain reaction (PCR) in RAW264.7 cells in vitro. Furthermore, the effects of CCK-oligosaccharides on spleen weight and mRNA expression levels of various cytokines (IL-6, IL-10, and TNF-α) in CY-treated mice were evaluated in vivo.

## 2. Results and Discussion

### 2.1. Effect of CCK-Oligosaccharides on MAPK Signaling Pathway of RAW264.7 Cells

**Figure 1:**
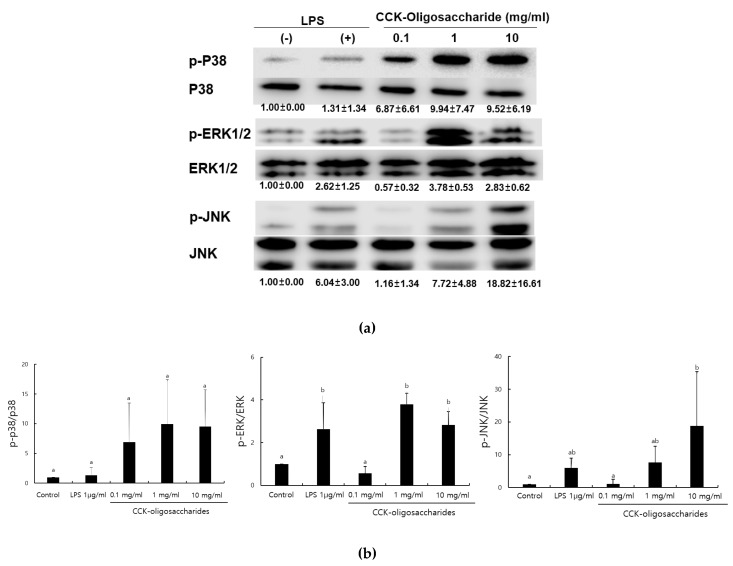
Effects of CCK-oligosaccharides treatment on MAPK signaling pathways in RAW264.7 macrophages. (**a**) Western blot images. Adherent RAW264.7 cells were incubated in medium with 0.1, 1, and 10 mg/mL CCK-oligosaccharides. The whole-cell lysates were prepared from the treated cells and the levels of both phosphorylated and non-phosphorylated MAPKs were analyzed by Western blot. Each blot is representative of three independent experiments and the numbers below blots indicate the relative expression level of MAPKs. (**b**) Quantification of Western blots. One-way ANOVA was used for comparison of group mean values, followed by Duncan’s multiple range test for significance of individual comparisons (*p* < 0.05). Different alphabet letters among groups represent statistically significant difference.

MAPKs include ERK, JNK, and P38, which control various immune responses, such as cell proliferation and differentiation, and play a particularly important role in macrophage activation [21,22]. Furthermore, CCK-oligosaccharides increase nitric oxide (NO) production in a dose-dependent manner [6]. When RAW264.7 macrophage cells were treated with CCK-oligosaccharides, the expression of P38, JNK, and ERK1/2 MAPK was determined (Figure 1). The expression of phosphorylated P38, ERK1/2, and JNK in 1 mg/mL CCK-oligosaccharide-treated RAW264.7 cells was higher than those in control-treated cells. Particularly, the expression of phosphorylated JNK increased in CCK-oligosaccharide-treated RAW264.7 cells in a dose-dependent manner. 

Alginate oligosaccharides enhance immunity in NF-κB and MAPK signaling pathways [7]. Glucogalactomannan isolated from *Cordyceps sinensis* mycelium [23], and the purified polysaccharide isolated from *Russula griseocarnosa*, an edible mushroom [24], also exert immunostimulating effects on NF-κB and MAPK signaling pathways.

### 2.2. Effect of CCK-Oligosaccharides on Phagocytic Activity of RAW264.7 Cells

**Figure 2:**
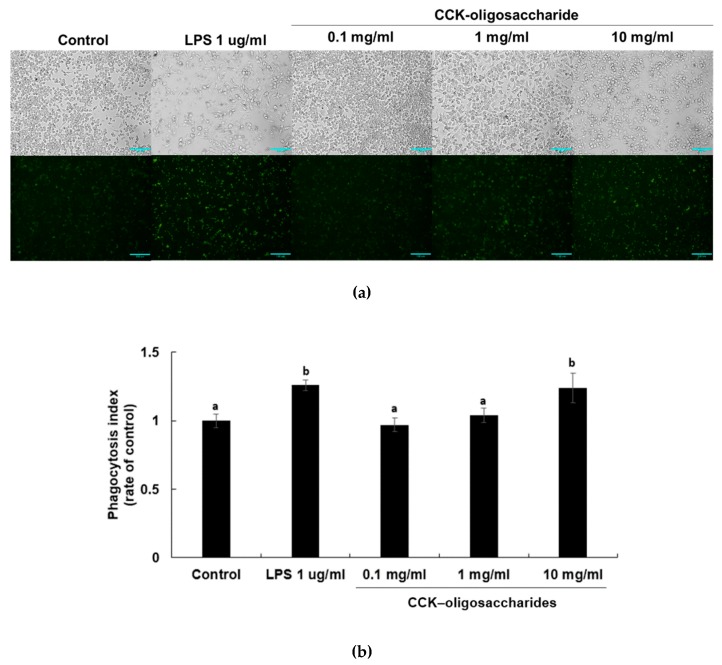
Effects of CCK-oligosaccharides on the phagocytic activity of RAW264.7 macrophages. (**a**) Fluorescent microscope images of RAW264.7 macrophage phagocytosis. Scale bar = 100 μm. (**b**) Quantification of rabbit IgG and FITC-coated latex bead uptake by RAW264.7 macrophages. One-way ANOVA was used for comparison of group mean values, followed by Duncan’s multiple range test for significance of individual comparisons (*p* < 0.05). Different alphabet letters among groups represent statistically significant difference. Each figure is representative of three independent experiments.

Phagocytosis is very important for innate and adaptive immunity as it stimulates defense against pathogens, tissue repair promoting, and chronic inflammation [25,26]. Activation of Fcγ receptors on the surface of the monocytes, macrophages, and neutrophils leads to broad-spectrum antimicrobial activities, including phagocytosis, secretion, cytokine synthesis, Ab-dependent cellular cytotoxicity, and generation of reactive oxidant species [27,28,29]. Effective phagocytosis by macrophages is caused by opsonization of pathogens, by Ig or complement proteins [18]. Phagocytosis of activated RAW264.7 macrophages has been determined by fluorescent IgG-coated latex beads [30]. To determine the effect of CCK-oligosaccharides on the phagocytic activity of RAW264.7 macrophages, FITC-conjugated IgG-coated latex beads were incubated with the cells after treatment (Figure 2).

The phagocytic index increased in a dose-dependent manner in CCK-oligosaccharide-treated RAW264.7 macrophages. The phagocytic indices of LPS-treated and 10 mg/mL CCK-oligosaccharide-treated cells were calculated to be 1.26 and 1.2, respectively. These results indicate that both LPS- and CCK-oligosaccharide-induction increased phagocytic activity, although CCK-oligosaccharide has lower phagocytic activity than LPS. The polysaccharide fraction of *Solanum nigrum* induces phagocytosis activity [30] and the purified polysaccharide isolated from *Caulerpa lentillifera* increases NO production, expression of P38 in MAPK signaling pathways, and phagocytosis activity, in a dose-dependent manner [31]. As the primary purpose of studying oligosaccharide produced by lactic acid bacteria is to examine the prebiotic activity of oligosaccharide, few studies have focused on the phagocytic activity of oligosaccharides, and this study is important as it shows that the oligosaccharides produced from *L. lactis* CCK 940 have a good phagocytic activity.

### 2.3. Effect of CCK-Oligosaccharides on Spleen Indices of CY-Induced Mice

The spleen is an important organ involved in nonspecific and specific immunity and spleen index reflects immune function and prognosis. Immunostimulation can increase the weight of the spleen [20,32]. 

**Figure 3:**
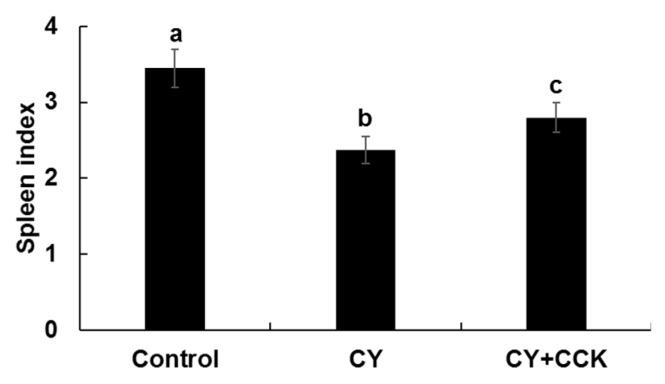
Spleen indices of CY-treated BALB/c mice with or without CCK-oligosaccharides. Control group (*n* = 6), no CY induction or CCK-oligosaccharides administration; CY group (*n* = 6), CY induction (80 mg/kg) on days 1–3 and saline administration on days 4–10; CY + CCK group (*n* = 6), CY induction (80 mg/kg) on days 1–3 and CCK-oligosaccharides administration (200 mg/kg) on days 4–10. One-way ANOVA was used for comparison of group mean values, followed by Duncan’s multiple range test for significance of individual comparisons (*p* < 0.05). Different alphabet letters among groups represent statistically significant difference.

Figure 3 shows that the spleen indices of the CY group mice was lower than those of the control and CY + CCK group mice, and spleen index had increased in the CY + CCK group mice. This result suggests that immunity decreases upon treating mice with CY. Treating mice with CCK-oligosaccharide increased the spleen indices. These findings indicate that CCK-oligosaccharides enhance immunity in immunosuppressed model mice. In other studies, the spleen indices of mice increased when CY-treated mice were exposed to polysaccharides of *Sophora subprosrate,* water-soluble polysaccharides of *Sargassum fusiforme*, and chito-oligosaccharides [20,33,34]. Many studies have shown that polysaccharides exert immunostimulating effects in CY-induced mice; however, only limited studies have focused on the immunostimulating effects of oligosaccharides obtained from lactic acid bacteria.

### 2.4. Effect of CCK-Oligosaccharides on mRNA Expression of Peritoneal Macrophages

Activated macrophages stimulate the production of various immunomodulatory cytokines, such as IL-6, IL-10, and TNF-α [35]. IL-6 is an important inflammatory and immune mediator that increases complement production and phagocytosis; it also regulates diverse cell functions as a growth factor, to enhance the proliferation and differentiation of neuronal cells, endothelial cells, T- and B- lymphocytes, and keratinocytes [36,37]. IL-10, an anti-inflammatory cytokine, is a Th2-type cytokine and tends to inhibit a broad range of inflammatory responses [38,39,40]. IL-10 is released under various conditions of immune activation by T cells, B cells, macrophages, and monocytes, and is known to be an important factor for supporting homeostasis of general immune responses [39,41]. It also exerts immunostimulatory effects, and it is involved in the activation of mast, T, B, and NK cells [42]. TNF-α is a powerful pro-inflammatory multifunctional cytokine, which is involved in inflammation, cell differentiation, proliferation, apoptosis, and promotion of immune cell functions [43,44,45].

**Figure 4:**
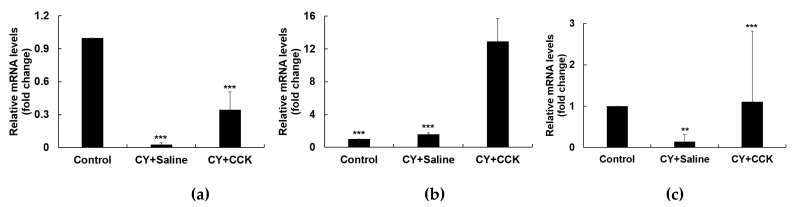
Effect of CCK-oligosaccharides on the mRNA expression levels of (**a**) IL-6, (**b**) IL-10, and (**c**) TNF-α in peritoneal macrophages in BALB/c mice. Group CY + Saline (*n* = 6), CY induction (80 mg/kg) on days 1–3 and saline administration on days 4–10; group CY + CCK (*n* = 6), CY induction (80 mg/kg) on days 1–3 and CCK-oligosaccharides administration (200 mg/kg) on days 4–10. Data are presented as the mean ± standard deviation of values of three independent experiments using six different mice. ** *p* < 0.05 and *** *p* < 0.001 vs. non-administered CY and CCK-oligosaccharides. The mRNA level in non-administered CY and CCK-oligosaccharides group was set to 1.

To determine the effects of CCK-oligosaccharides on CY-induced immunosuppression, peritoneal macrophages of BALB/c mice were isolated and mRNA expression was evaluated. In peritoneal macrophages, the intraperitoneal treatment of CY inhibited the expression of cytokines. The administration of CCK-oligosaccharides increased the expression of IL-6, IL-10, and TNF- α compared with only those in the CY-treated mice (Figure 4a–c).

When mice were administered with CCK-oligosaccharides at a concentration of 200 mg/kg, mRNA expression levels of IL-6, IL-10, and TNF-α were 10 times higher than those in mice only administered with CY. These results show that CCK-oligosaccharide administration led to the enhancement of immune functions in an immunosuppressed mice model. Curdlan oligosaccharides from *Alcaligenes faecalis* var. *myxogenes* activate MAPK and NF-κB pathways and improve immunostimulatory activity in CY-induced imuunosuppressed mice [46]. Polysaccharides of *Lonicera japonica*, a typical Chinese herbal medicine, and wheat bran-derived polysaccharides markedly promote the serum cytokine levels [47,48], and polysaccharides derived from *Stichopus japonicus* (sea cucumber), up-regulate the expression of cytokines, such as IL-1β, IL-4, IL-6, IL-10, TNF-α, and IFN-γ, in the CY-induced mice [49]. As CCK-oligosaccharides have an immunostimulating activity, it can be used as an ingredient for functional foods to regulate immune responses. Even though gluco-oligosaccharides are considered safe, the structural changes by the intestinal microflora might result in undesirable effects. Correspondingly, the safety issue of oligosaccharides would be considered in the development of functional foods.

## 3. Materials and Methods

### 3.1. Preparation of CCK-Oligosaccharides

CCK-oligosaccharides were purified by the protocol described in our previous study [6]. Briefly, *L. lactis* CCK940 was cultured in Lactobacilli MRS broth (BD, Franklin Lakes, NJ, USA) at 30 °C for 20 h. The culture was inoculated to LM media [50], supplemented with 9.6% (*w*/*v*) sucrose and 7.4% (*w*/*v*) maltose, and incubated at 30 °C for 9 h. The culture supernatants were concentrated and separated by gel permeation chromatography (GPC; Bio-gel P2; Bio-Rad, Hercules, CA, USA). The oligosaccharide fractions were collected and lyophilized (SunilEyela, Seongnam, Korea) to determine their immunological effects.

### 3.2. Cell Culture

A murine macrophage cell line, RAW264.7, was cultured in Dulbecco’s Modified Eagle’s Medium (DMEM, Gibco, NY, USA), and supplemented with 10% (*v*/*v*) bovine serum (Gibco) and 1% (*v*/*v*) penicillin–streptomycin (Gibco) at 37 °C in a 5% CO_2_ incubator (Thermo Fisher Scientific, Waltham, MA, USA).

### 3.3. Western Blot

RAW264.7 cells were cultured in 6-well plates (2 × 10^6^ cells/well) for 24 h with 5% CO_2_ at 37 °C Total protein from the RAW264.7 cells was extracted using RIPA buffer (Cell Signaling Technology, Danvers, MA, USA), containing phosphatase inhibitor cocktail (BioVision, Milpitas, CA, USA). Protein concentration was measured using the Pierce BCA Protein Assay Kit (Thermo Fisher Scientific, Waltham, MA, USA). Proteins were separated by SDS–PAGE (12.5%) and transferred to polyvinylidene difluoride membranes (Amershan Hybond, GE Healthcare, Chicago, IL, USA). The membranes were blocked with 5% skim milk in Tris-buffered saline containing 0.1% Tween 20, incubated with primary antibodies against P38, phospho-P38, ERK1/2, phospho-ERK1/2, JNK, phospho-JNK (1:1000, Cell Signaling Technology, Danvers, MA, USA) at 4 °C for overnight, and again incubated with secondary antibodies for 2 h at 20 °C. The chemiluminescent signals emitted after incubation with secondary antibodies were detected with EzWestLumi, plus chemiluminescent substrate (ATTO Corporation, Tokyo, Japan) and Odyssey LCI Image software (LI–COR Biosciences, Lincoln, NE, USA).

### 3.4. Phagocytosis Assay

RAW264.7 cells were cultured in 24-well plates (2 × 10^5^ cells/well) for 24 h with 5% CO_2_ at 37 °C Cells were treated with 0.1, 1.0, and 10.0 mg/mL CCK-oligosaccharides, or 1 μg/mL lipopolysaccharide (LPS) as a control, and incubated at 37 °C in a 5% CO_2_ incubator for 24 h. Each group was treated with specific FITC-conjugated rabbit IgG-coated latex beads (Cayman Chemical, Ann Arbor, MI, USA) to determine whether CCK-oligosaccharides affected the phagocytic activity of fluorescence particles in RAW264.7 cells. Random sites were photographed, and Live Imaging software was used to acquire real-time microscope (Nikon ECLIPSE Ti, Nikon Instruments Inc., Tokyo, Japan) images over a period of 24 h. Imaging software files were exported and analyzed in MetaMorph software version 7.8.9.0 (Molecular device, Sunnyvale, CA, USA).

### 3.5. Animal Experiments

#### 3.5.1. Animals

The study was performed on 6-week old male BALB/c mice, with medium weight 23 ± 2 g, obtained from Orient Bio Inc. (Suwon, Korea). The mice were housed with sterile bedding under 12/12 h light/dark schedule in a temperature- and humidity-controlled room, with ad-libitum access to food and water. Animal experiments were conducted in strict accordance with the recommendations in the Guide for the Care and Use of Laboratory Animals of the National Institutes of Health. The protocol was approved by the Institutional Animal Care and Use Committee (IACUC) of Woojung BSC, Inc. (Approval Number WJIACUC 20181002–3–34) in conjunction with the Advanced Institute of Convergence Technology, Seoul National University.

#### 3.5.2. Induction of Immunosuppressed Murine Model and Treatment Protocols

Mice were grouped in three groups (*n* = 8 in each group). The control group mice were fed sterile saline solution on days 4–10. Mice of the second group (CY group) were intraperitoneally injected with 80 mg/kg CY on days 1–3 (Sigma Aldrich, St. Louise, MO, USA) as an immunosuppressant and fed sterile saline solution on days 4–10. The third group mice (CY + CCK group) were intraperitoneally injected with 80 mg/kg CY on days 1–3 and fed 200 mg/kg CCK-oligosaccharide on days 4–10. On day 11, the mice were euthanized by carbon dioxide exposure.

#### 3.5.3. Calculation of Spleen Indices

Mice were sacrificed on day 11 and the spleen of each mouse was aseptically removed and weighed. The spleen index was calculated using the following formula:

Spleen index = Spleen weight (mg)/Body weight (g)(1)

#### 3.5.4. Isolation of Mouse Peritoneal Macrophages

For isolation of peritoneal macrophages, mice were intraperitoneally injected with 10 mL cold phosphate buffered saline solution (PBS) after scarification. After a soft abdominal massage, the peritoneal PBS was collected to sterilize tubes. The collected PBS was centrifuged at 200× *g* for 10 min and peritoneal macrophages were isolated using RBC lysis buffer (Sigma Aldrich, St. Louise, MO, USA). Peritoneal macrophages were cultured in RPMI 1640 media (HyClone, GE Healthcare, Chicago, IL, USA) at 37 °C, in a 5% CO_2_ incubator.

#### 3.5.5. RNA Extraction and cDNA Synthesis

The expression levels of cytokines were measured to confirm the immunostimulating effect of the CCK-oligosaccharides on mouse peritoneal macrophages, and cells were cultured in a 12-well plate at 37 °C in a 5% CO_2_ incubator. RNA was isolated using easy-BLUETM Total RNA Extraction kit (iNtRON Biotechnology, Inc., Seongnam, Korea) from the cultured cells. Transcriptor First Strand cDNA Synthesis Kit (Roche, Basel, Switzerland) was used to synthesize cDNA from the isolated RNA, and the synthesized cDNA was used for real-time PCR (LightCycler96, Roche, Basel, Switzerland), using the FastStart Essential DNA Green Master Kit (Roche, Basel, Switzerland).

#### 3.5.6. Real-Time PCR

IL-6, IL-10, and TNF-α cytokine genes were amplified. GAPDH was used as an endogenous control gene and the expression levels of cytokine mRNA relative to those of the GAPDH mRNA were analyzed using the 2^-ddCT^ method. The sequences of the primers used in this study are listed in Table 1.

### 3.6. Statistical Analysis

Data are expressed as mean ± standard deviation (SD) of values from triplicate experiments. Statistical analyses were performed using SPSS 23 (SPSS Inc., Chicago, IL, USA). Statistical significance between groups was determined by a paired *t*-test for repeated measures. Data with *p* < 0.05, *p* < 0.01, and *p* < 0.001 were considered statistically significant. One-way ANOVA was used for comparison of group means, followed by Duncan’s multiple range test for significance of individual comparisons (*p* < 0.05).

## 4. Conclusions

This study demonstrated that CCK-oligosaccharides have a potent immunomodulating activity, which predominantly results from CCK-oligosaccharide-induced upregulation of phagocytic activity and MAPK signaling pathways in RAW264.7 macrophages. These activities were due to the activation of major signaling proteins, such as P38, JNK, and ERK, as well as TNF-α. In vivo study also showed that CCK-oligosaccharides can promote immune function in CY-induced immunosuppressed mice, presumably due to the activation of the spleen. To the best of our knowledge, this is the first study to report the immunostimulatory activity of gluco-oligosaccharides produced from *Leuconostoc* sp. and demonstrate that CCK-oligosaccharides may be used as an agent developed from functional foods to regulate immune responses.

## Figures and Tables

**Table 1:** Cytokine primer sequences for real-time PCR.

Gene		Sequence
**GAPDH**	Forward	5′―ATC CCA TCA CCA TCT TCC AG―3′
	Reverse	5′―CCT GCT TCA CCA CCT TCT TG―3′
IL-6	Forward	5′―CAA GAG ACT TCC ATC CAG TTG C―3′
	Reverse	5′―TTG CCG AGT TCT CAA AGT GAC―3′
IL-10	Forward	5′―TGC TAT GCT GCC TGC TCT TA―3′
	Reverse	5′―GGC AAC CCA AGT AAC CCA TA―3′
TNF-α	Forward	5′―ATG AGC ACA GAA AGC ATG ATC CG―3′
	Reverse	5′―CCA AAG TAG ACC TGC CCG GAC TC―3′
